# 5-Azacytidine Promotes an Inhibitory T-Cell Phenotype and Impairs Immune Mediated Antileukemic Activity

**DOI:** 10.1155/2014/418292

**Published:** 2014-03-13

**Authors:** Thomas Stübig, Anita Badbaran, Tim Luetkens, York Hildebrandt, Djordje Atanackovic, Thomas M. C. Binder, Boris Fehse, Nicolaus Kröger

**Affiliations:** ^1^Department of Stem Cell Transplantation, University Medical Center Hamburg-Eppendorf, Martinistrasße 52, 20246 Hamburg, Germany; ^2^Department of Oncology/Hematology, University Medical Center Hamburg-Eppendorf, Martinistrasße 52, 20246 Hamburg, Germany; ^3^Department for Transfusion Medicine, University Medical Center Hamburg-Eppendorf, Martinistrasße 52, 20246 Hamburg, Germany

## Abstract

Demethylating agent, 5-Azacytidine (5-Aza), has been shown to be active in treatment of myeloid malignancies. 5-Aza enhances anticancer immunity, by increasing expression of tumor-associated antigens. However, the impact of 5-Aza immune responses remains poorly understood. Here, T-cell mediated tumor immunity effects of 5-Aza, are investigated *in vitro* and *in vivo*. T-cells from healthy donors were treated with 5-Aza and analyzed by qRT-PCR and flow cytometry for changes in gene expression and phenotype. Functionality was assessed by a tumor lysis assay. Peripheral blood from patients treated with 5-Aza after alloSCT was monitored for changes in T-cell subpopulations. 5-Aza treatment resulted in a decrease in CD8+ T-cells, whereas CD4+ T-cells increased. Furthermore, numbers of IFN-**γ**+ T-helper 1 cells (Th1) were reduced, while Treg-cells showed substantial increase. Additionally, CD8+ T-cells exhibited limited killing capacity against leukemic target cells. *In vivo* data confirm the increase of Treg compartment, while CD8+ T-effector cell numbers were reduced. 5-Aza treatment results in a shift from cytotoxic to regulatory T-cells with a functional phenotype and a major reduction in proinflammatory Th1-cells, indicating a strong inhibition of tumor-specific T-cell immunity by 5-Aza.

## 1. Introduction 

Methylation plays a central role in the epigenetic regulation of gene expression [1]. Cancer cells in particular use hypermethylation to switch off a vast number of genes, responsible for growth inhibition, differentiation, and apoptosis [2]. Treatment induced differentiation in myeloid malignancies was reported to exhibit substantial clinical benefit and, accordingly, demethylating drugs like 5-Azacytidine (5-Aza) have been introduced into the therapy of myelodysplastic syndrome (MDS) [3] and acute myeloid leukemia (AML) [4].

After cellular uptake, 5-Aza is phosphorylated to 5-aza-2′-deoxycytidine-5′-triphosphate and subsequently is incorporated into the DNA, to inhibit the methylating enzyme DNA methyltransferase [5]. Supplementary to its effects on genes responsible for cell growth and differentiation, 5-Aza was found to upregulate tumor-associated antigens, such as cancer-testis antigens (CTA), potentially augmenting immune recognition of malignancies [6–8]. Several small studies have recently introduced simultaneous application of 5-Aza combined with donor lymphocyte infusions in AML patients [9–12]. However, due to its broad mechanism of action, 5-Aza may have an impact on the quality of antitumor immunity in various ways, as reported by a recent study describing its immunosuppressive properties in mice [13].

Like most eukaryotic cells, CD4+ T-cells use epigenetic mechanisms to regulate lineage commitment [14]. Particularly transcription factor FoxP3, as a master regulator of regulatory T-cells [15], has been described to be strongly regulated by methylation [16, 17]. Even though our knowledge on epigenetic regulation in CD8+ T-cells is still limited, memory function and Interferon gamma (IFN-*γ*) production in murine CTL also seems to be controlled by methylation [18–20]. In this study, we investigated the effects of 5-Aza as a demethylating agent on the phenotype and function of human T-cells* in vitro *and* in vivo*.

## 2. Patients, Materials, and Methods

### 2.1. Cells and Cell Culture

T-cells were isolated from buffy coats from healthy donors (*n* = 10). CD3+, CD4+, and CD8+ T-cells were sorted using the MACS system (Miltenyi, Bergisch Gladbach, Germany). Purity of CD3+ (>98%) and CD4+ and CD8+ T-cells (>96%) was determined by flow cytometry. T-cells were stimulated with CD3/CD28 beads (Invitrogen, Carlsbad, USA) and cultured in RPMI (Gibco, Karlsruhe, Germany) with 15% autologous, heat-inactivated, plasma, 1% Penicillin/Streptomycin (Gibco, Karlsruhe, Germany), and 90 U IL2 (Proleukin, Novartis, Germany).

Cell lines HL60 and K562 (DSMZ, Braunschweig, Germany) were cultured in RPMI medium, 10% fetal bovine serum, and 1% Penicillin/Streptomycin (both Gibco, Karlsruhe, Germany).

### 2.2. Chemicals and Antibodies

5-Azacytidine was obtained from Sigma-Aldrich (Munich, Germany) and used at a final concentration of 5 *μ*M or 20 *μ*M. All flow cytometry experiments were performed using a FACS Canto II (BD Bioscience, Heidelberg, Germany). For flow cytometry the following antibodies were used: anti-CD3-PE, anti-CD4-FITC, anti-CD8-APC, anti-CD25-PE, anti-CD45-FITC, anti-CD45RO-PE, anti-CD45RA-FITC, anti-CD62L-PE, anti-CCR7-PE, anti-HLA-DR-APC, anti-Granzyme-PE antibodies (obtained from BD Bioscience) anti-CD127-PC5, anti-IFN*γ*-PE/Cy7, anti-IL17-APC, anti-IL4-FITC, and anti-FoxP3-APC antibodies obtained from eBioscience (San Diego, USA).

### 2.3. RNA Isolation, cDNA Synthesis, and Quantitative RT-PCR

RNA was isolated using the Qiagen RNeasy kit (Qiagen, Hilden, Germany). 1 *μ*g of total RNA was used for RT reaction using the cDNA superscript kit (Bio Rad, Munich, Germany).

Quantitative RT-PCR was performed using SYBR Green (Fermentas, St. Leon-Rot, Germany) and primers for the following transcripts:* p15, p16, p21, FOXP3, TBET1, GATA3, RORgt, IL-10, TGF-*β*,* and* GAPDH* were obtained from Qiagen (Hilden, Germany). PCR was carried out in a Chromo 4 cycler (Bio Rad, Munich, Germany). Gene expression was normalized to* GAPDH* expression and relative gene expression was calculated by using the ΔΔCT method normalized to cDNA of Jurkat cells.

### 2.4. Flow Cytometric Analysis of Intracellular Cytokines

For the analysis of intracellular cytokine expression T-cells were stimulated with phorbol myristate acetate (PMA), Ionomycin for 1 hour and Brefeldin A for 4.5 hours. All chemicals were obtained from Sigma-Aldrich (Munich, Germany). Cells were harvested and prepared for analysis using the Cytofix/Cytoperm kit (BD Bioscience, Heidelberg, Germany). For intracellular cell staining the following antibodies were used: anti-IL4-FITC, anti-IL17-APC, anti-IFN*γ*-PECy7, and anti-FOXP3-APC, obtained from eBioscience (San Diego, USA) and anti-Granzyme-PE (BD Bioscience, Heidelberg, Germany).

### 2.5. LDH Cytotoxicity Assay

Cells were treated with or without 5-Aza for an additional 48 h. Viable T-cells were determined by trypan blue exclusion and 50.000 T-cells were cocultured with target cell lines (HL60 and K562) in different ratios for example, 1 : 5, 1 : 10, and 1 : 20. Cells were cocultured for 4 h and specific cytotoxicity was measured using the CytoTox 96 assay (Promega, Madison, USA), according to the manufacturer's instructions. In brief pretreated T-cells were cocultured with target cells, for additional 4 hours. The assay measures the released LDH in culture supernatants in a 30-minute coupled enzymatic assay, which results in conversion of a tetrazolium salt into a red formazan product. The amount of color formed is proportional to the number of lysed cells. As additional controls to untreated cells we used K562 cells for spontaneous lysis. Cell death was calculated according to the company provided formula. All cytotoxicity experiments were performed in triplicates and the mean value was used for further analysis.

### 2.6. Patients

A total of 3 patients (3 male) were treated after allogeneic stem cell transplantation with 5-Azacytidine. The median age was 60 years (range 57–63 years). All three patients received stem cells from a matched unrelated donor. Before stem cell transplantation 2 patients had received a reduced intensity conditioning and one patient a myeloablative conditioning. No patient developed an acute GvHD after transplantation or during 5-Aza treatment. Patients' characteristics are shown in Supplementary Table  1 available online at http://dx.doi.org/10.1155/2014/418292.

All patients and healthy donors agreed to biological material donation by informed written consent under an approved protocol from the “Ärztekammer Hamburg.”

### 2.7. Treatment

Treatment with 5-Azacytidine (Vidaza, Celgene, USA; 5-Aza) was started between 66 and 127 days after allogeneic stem cell transplantation, when patients showed a minimal residual disease or a relapse (defined as mixed chimerism or evidence of a molecular or cytogenetic marker, relapse was defined as blast count > 10% in peripheral blood and bone marrow). Minimal treatment criteria were white blood cell count > 3.0 × 10^9^/L and platelet > 20 × 10^9^/L. Azacytidine was given subcutaneously 100 mg/m^2^ each day, day 1–5. The next cycle started at day 30, with a planned total of 2 cycles. Two patients received 2 cycles and treatment continuously; in one patient treatment was stopped because of progressive disease after 1 cycle.

### 2.8. Statistics

Data were analyzed using the GraphPad Prism 5 software (GraphPad Software, LaJolla, USA). Student's *t*-test was used to compare mean values of at least three independent experiments or values obtained from the three different patients. *P* < 0.05 was considered statistically significant.

## 3. Results

### 3.1. 5-Azacytidine Inhibits CD8+ T-Cell Growth and Correlates with Overexpression of Cell Cycle Inhibitor* p15*

**Figure 1:**
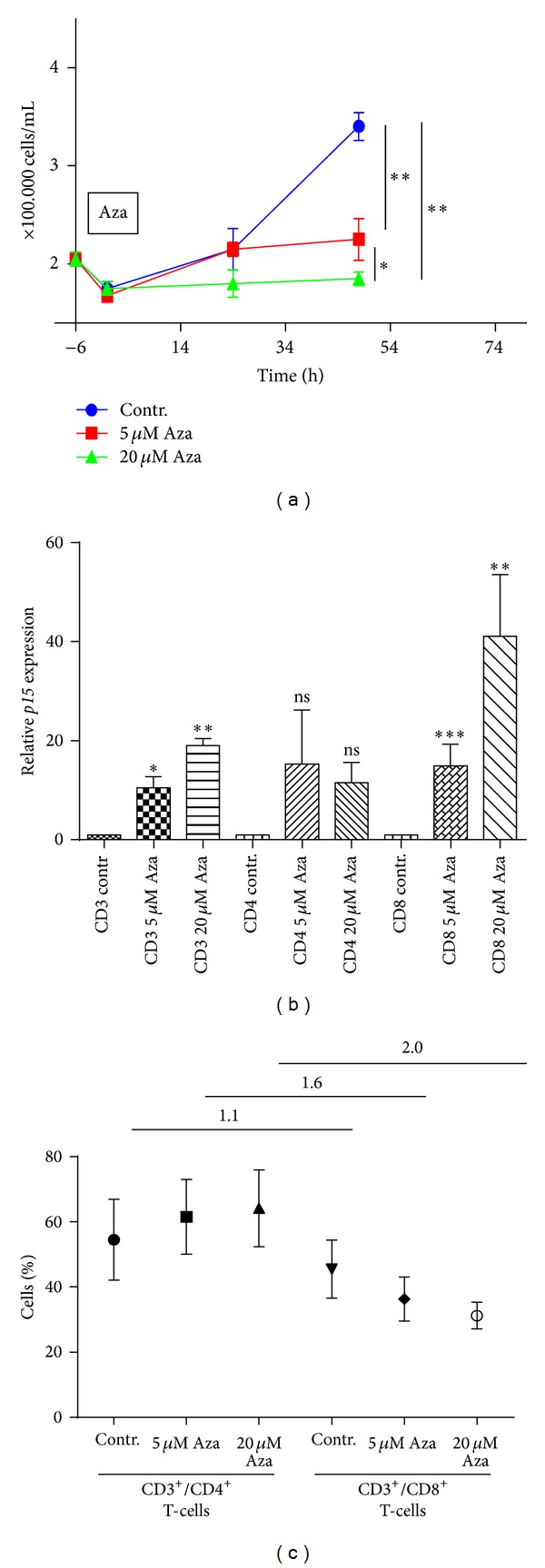
5-Azacytidine reduces T-cell proliferation mainly by inhibition of CD8 T-cell proliferation by* p15* upregulation. (a) T-cells were isolated from buffy coats and cultured for one week in presence of IL-2. 12 h before 5-Aza treatment cells were seeded in fresh medium. T-cells were treated with 5 *μ*M or 20 *μ*M 5-Aza or left untreated as control. Numbers of viable cells were determined every 12 h, over a period of 72 h by Trypan blue exclusion. Depicted results show mean value of triplicates. (*P < 0.05, **P < 0.005). (b) CD3, CD4, and CD8 T-cells were sorted and treated with (5 *μ*M or 20 *μ*M) or without 5-Aza.* p15* mRNA levels of the different subsets were analyzed by qRT-PCR. Data show mean values of triplicates. (*P < 0.05, **P < 0.005, not significant (n.s.)). (c) After 5-Aza for 48 h T-cell subsets were analyzed by FACS with mAB against CD3, CD4, and CD8. CD4/CD8 ratios were indicated for each treatment group. Data represent mean value of four independent experiments with SD.

5-Aza is known to influence the growth of tumor cells by upregulation of cell cycle inhibitors [21]. We therefore asked whether 5-Aza would also impact the growth of T-cells. Cells were treated for 72 h with different concentrations of 5-Aza, while cell viability was assessed every 12 hours (Figure 1(a)). We found that 5-Aza treatment led to a significant decrease of T-cell growth after 48 h in a dose-dependent manner. By screening mRNA expression of key cell cycle inhibitors we found that* p15* was strongly upregulated, especially after treatment with the higher 5-Aza concentration (Figure 1(b)).

To determine if T-cell subsets react uniformly to 5-Aza treatment, we compared the compartment-specific response of CD4+ to CD8+ T-cells. After 48 h of 5-Aza treatment we observed an increasing CD4/CD8 ratio (Figure 1(c)), which might be caused either by a proliferation advantage of CD4+ T-cells or by a stronger inhibition of CD8+ T-cell growth. Analysis of the expression of key cell cycle inhibitory genes in both subsets indicated an increase of* p15*, originating predominantly from CD8+ T-cells (Figure 1(b)). These data suggest that CD8+ T-cells are more susceptible to cell cycle inhibition by 5-Aza, thus promoting a shift towards a CD4+ T-cell phenotype.

### 3.2. Induction of Regulatory T-Cells by 5-Azacytidine

**Figure 2:**
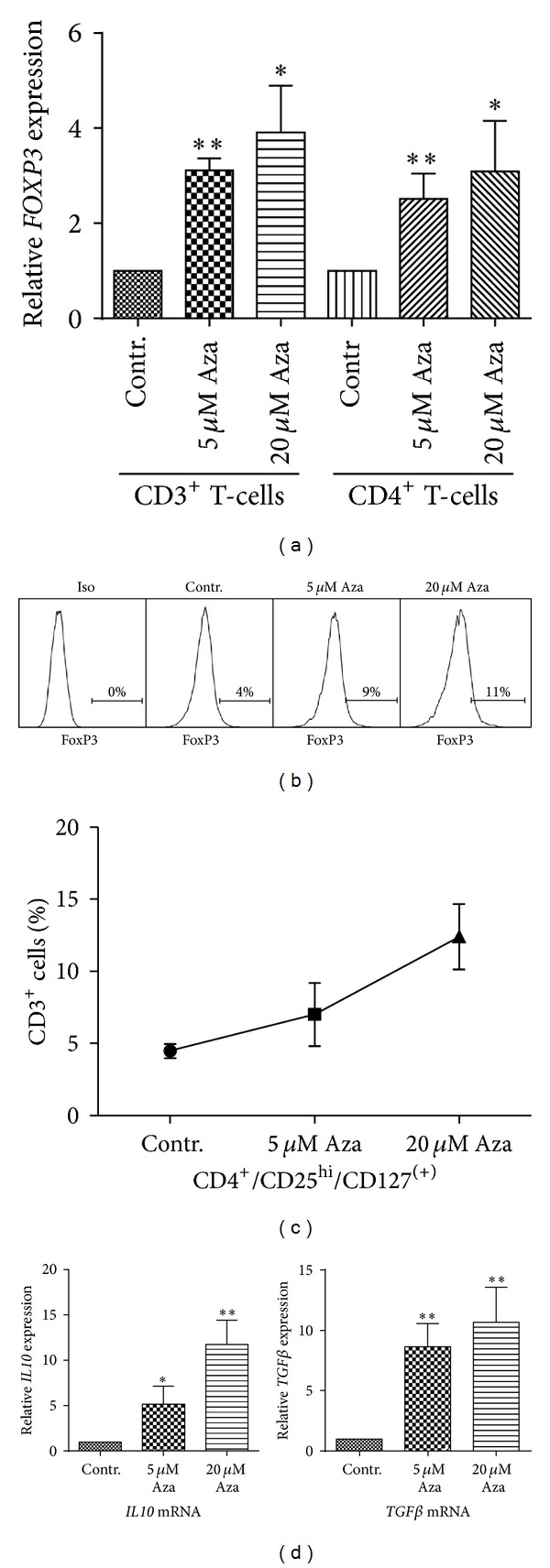
Treatment with 5-Aza induces FOXP3+ Treg and upregulates immunomodulatory cytokines. (a) CD3+ and CD4+ T-cells were isolated and cultured for one week in the presence of IL2. Thereafter, cells were treated with the indicated dosages of 5-Aza. After 48 h mRNA was isolated and* FOXP3* expression levels were analyzed in the CD3+ and CD4+ T-cell subset. Data show mean value with SD. (*P < 0.05, **P < 0.005). (b) T-cells were analyzed by FACS after the described treatment with or without 5-Aza for the expression of FoxP3. CD4+/CD25+/FoxP3+, triple positive cells were considered as Tregs. A representative example of three independent experiments is shown. (c) The marker combination CD4+/CD25hi/CD127lo was used to confirm the results of (b). Data show mean value with SD of three different experiments. (d) mRNA of 5-Aza treated or control CD3+ T-cells were isolated and mRNA levels of* IL10* and* TGF-*β** were measured. Data shown as fold pattern gene induction. (*P < 0.05, **P < 0.005).

Expression of the main regulatory T-cell transcription factor* FOXP3* is strongly regulated by DNA methylation [16, 17]. We, therefore, assessed whether treatment with the demethylating agent 5-Aza would lead to a change in* FOXP3* expression. As hypothesized, qRT-PCR revealed a 3–3.5-fold upregulation of* FOXP3* after 5-Aza treatment of CD3+ T-cells (Figure 2(a)). Accordingly, we observed an approximate threefold increase in the CD4+CD25+FOXP3+, Treg fraction at the highest 5-Aza dosage (Figure 2(b)). This was confirmed by staining of the alternative Treg phenotype CD4+CD25hiCD127lo (Figure 2(c)). Our data strongly suggest that the overall shift from CD8+ to CD4+ T-cells occurs predominantly in the CD4+ regulatory population.

Treg function is mainly based on the production of inhibitory cytokines which alter the activity of effector T-cells. We, therefore, investigated mRNA transcription of two of the major inhibitory cytokines,* IL-10* and* TGF-*β*,* and found that both cytokines were significantly upregulated after 5-Aza treatment in a dose-dependent manner. These findings indicate that 5-Aza treatment increases not only the number of Treg cells, but also the production of major inhibitory cytokines.

### 3.3. Treatment with 5-Azacytidine Reduces the Killing Capacity of CD8+ T-Cells Independent of Treg Inhibition

**Figure 3:**
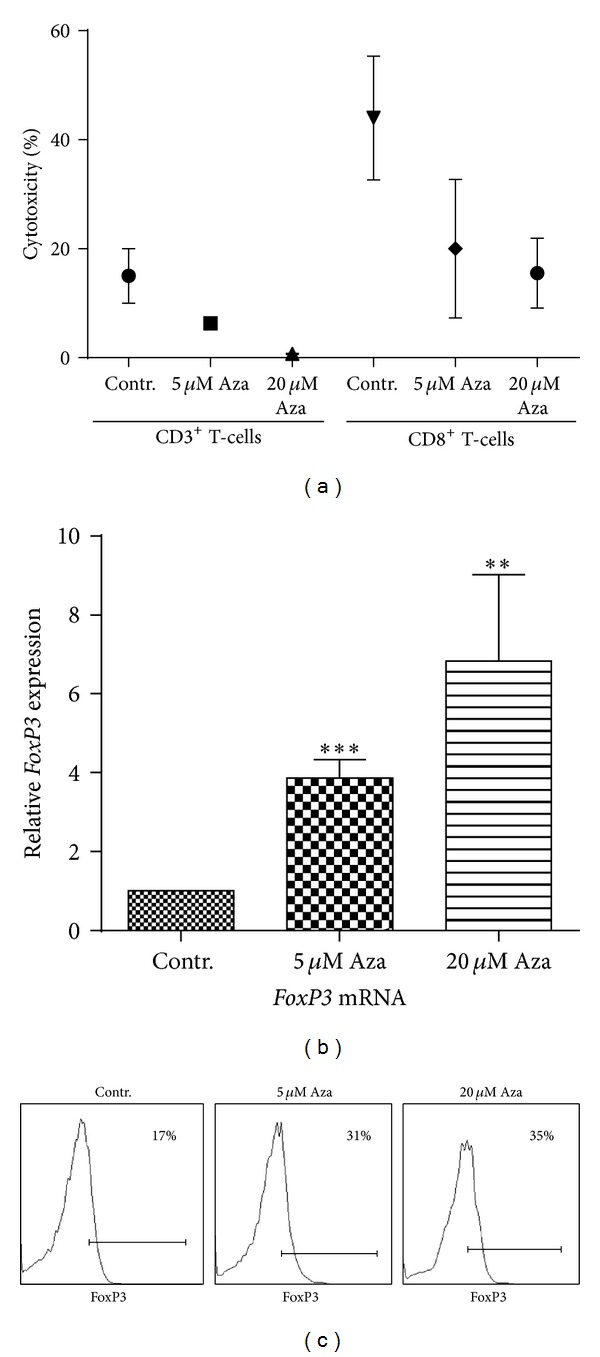
CD8 T-cells show reduced cytotoxic function after 5-Aza treatment and upregulation of* FOXP3*. (a) CD3+ or CD8+ T-cells were isolated and treated with 5 *μ*M or 20 *μ*M or without 5-Aza for 48 h. Thereafter, T-cells were resuspended in fresh medium and cocultured with the target cell line HL60 for 4 h. LDH release was used as marker for cell death. Specific cytotoxicity was calculated as described by the manufacturer. Four independent experiments (all with an E : T ratio 10 : 1) are shown. (b) CD8+ T-cells were sorted and treated with 5-Aza (5 *μ*M and 20 *μ*M) for 48 h. mRNA levels of* FOXP3* in CD8 cell compartment were assessed by qRT-PCR. (*P < 0.05). (c) CD8+ T-cells were analyzed after treatment with or without 5-Aza, for their intracellular expression of FoxP3. A representative of three different experiments is shown.

Cytotoxic T-cells have the ability to recognize and eradicate malignant cells [22]. However, Treg cells are able to mitigate this effect* in vivo* and may play a central role in tumor immune escape [23]. We, therefore, assessed whether the observed increase of Treg cells after 5-Aza treatment influences the ability of effector T-cells to kill tumor cells. We treated CD3+ T-cells for 48 h with 5-Aza and subsequently cocultured them with leukemic cell line, HL60. The percentage of specific killing was measured using an LDH release assay. We found a strong reduction in the killing capacity of CD3+ T-cells against HL60 (Figure 3(a)). In order to verify the potential effect of Treg cells on CD8+ cytotoxic effector T-cells, we repeated the assay, this time using isolated CD8+ T-cells at high (>98%) purity, thus excluding contamination with relevant numbers of Treg cells. We, once more, observed a clear reduction of the killing capacity by 5-Aza. As demethylating treatment appeared to directly affect the function of CD8+ T-cells, we next speculated on potential explanations for this observation. In this aspect, CD8+ cells, so-called suppressor T-cells, which are associated with a less cytotoxic phenotype, have been reported to express* FOXP3*. Our results indicate that CD8+ T-cells upregulate* FOXP3* mRNA up to 4-fold, after 5-Aza treatment (Figure 3(b)). This increase is significantly higher than in the observed of the CD4+ T-cell compartment (*P* = 0.04) and was confirmed by an increase in the numbers of CD8+FOXP3+ cells measured by flow cytometry (Figure 3(c)). We further analyzed the CD8+ T-cells for their expression of granzyme strikingly to the observed reduction of CD8+ T-cell mediated tumor cell killing we found that granzyme production was reduced after 5-Aza treatment (Supplementary Figure S1).

### 3.4. 5-Azacytidine Treatment Leads to a Reduction of Proinflammatory Th1 Cells

**Figure 4:**
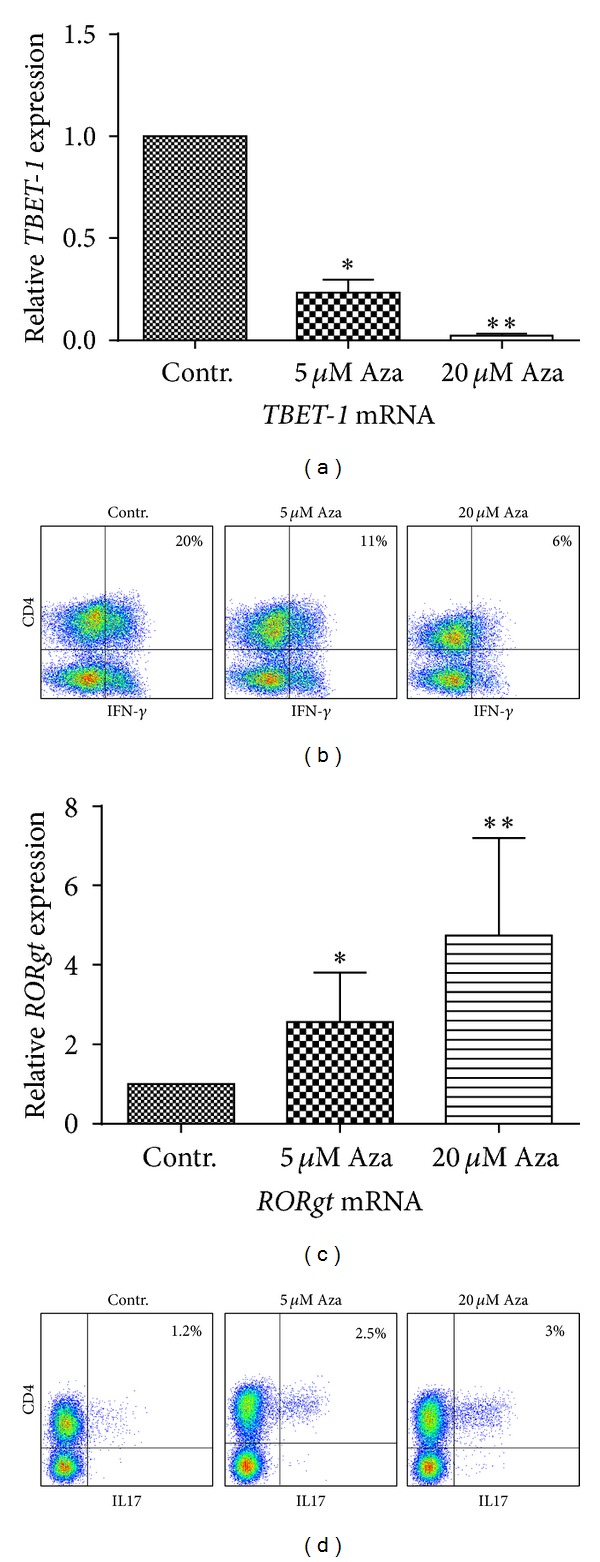
*In vitro* treatment with Azacytidine has different influence on TH1 and TH17 cells. (a) CD4+ T-cells were treated with or without 5-Aza for 48 h. mRNA was isolated and the expression of* TBET1* was analyzed by qRT-PCR. Data are shown as mean value with SD. (*P < 0.05, **P < 0.005). (b) CD4+ T-cells were analyzed for their expression of IFN-*γ*, after T-cell treatment with or without 5-Aza and additional stimulation with PMA and ionomycin in the presence of brevedinA for 4.5 h. IFN-*γ* expression was analyzed by FACS. A representative example of three independent experiments is shown. (c) After sorting, CD4+ T-cells were treated with or without 5-Aza for 48 h, and mRNA was isolated. Expression of* ROR*γ*t* was analyzed by qRT-PCR. Data are shown as mean value with SD. (*P < 0.05, **P < 0.005, not significant (n.s.)). (d) Similar to the aforementioned IFN-*γ* staining, CD4+ T-cells were analyzed for their expression of IL17 after 5-Aza treatment. A representative sample of three independent experiments is shown.

Modulation of the lineage-specific differentiation of T-helper cell subsets represents one of the major contributions of Treg cells to peripheral tolerance [15]. Development of CD4+ T-cell subsets Th1, Th2, and Th17 is further mediated by epigenetically controlled hallmark transcription factors* TBET1*,* GATA3*, and* ROR*γ*t*, respectively [14]. In order to determine the influence of 5-Aza on T-helper differentiation, we analyzed purified CD4+ T-cells treated with 5-Aza for 48 h and measured the expression of specific transcription factors and cytokines associated with each subset function, by qRT-PCR and flow cytometry. We did not observe statistically significant changes in* GATA3* mRNA expression (data not shown), suggesting negligible effects of 5-Aza on Th2 development. In contrast, we found a significant reduction in* TBET1 *mRNA (Figure 4(a)). Moreover, we observed a dose-dependent decrease of cells producing IFN-*γ* protein (Figure 4(b)) indicating impaired Th1 differentiation. Conversely,* ROR*γ*t* mRNA levels (Figure 4(c)) and the frequency of cells expressing IL-17 protein (Figure 4(d)), both associated with Th17 development, were elevated.

### 3.5. 5-Azacytidine Promotes a Shift from Memory to Naïve T-Cells

**Figure 5:**
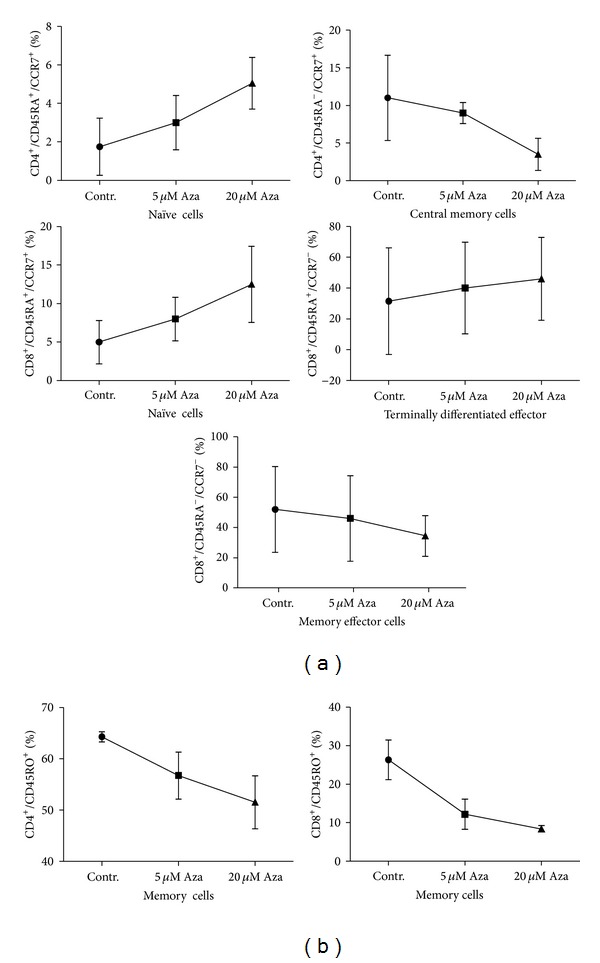
Azacytidine inhibits memory T-cells while naïve T-cell number is not affected. CD4+ and CD8+ T-cells were isolated and treated with or without the discussed 5-Aza concentrations. 48 h after treatment, cells were assessed for the expression of CD4, CD8, CD45RA, CCR7, and CD45RO. A representative sample and the gating strategy are shown in Supplementary Part (Supplementary Figure S2). (a) Cells expressing both (CD45RA+/CCR7+) were considered to be naïve T-cells. (b) Cells expressing the CD45RO antigen were considered to be memory T-cells. Data were shown as mean with standard deviation, a summary of three independent experiments is shown. Plots show a percentage of gated T-cells.

As IFN*γ* is crucial for the development of memory T-cells [24] we assessed whether 5-Aza is associated with a change in the memory phenotype. In CD3+ T-cells treated with 5-Aza for 48 h, we first investigated the expression of memory marker CD45RO and the naïve marker CD45RA. We observed a substantial reduction of CD45RO+ memory cells but an increase of naïve cells in both the CD4+ and the CD8+ T-cell compartment (Figure 5). We next costained with CCR7 to further differentiate between naïve cells (CD45+/CCR7+), terminally differentiated effector cells (CD45+/CCR7−), central memory T-cells (CD45−/CCR7+), and effector memory cells (CD45−/CCR7−). CD4+ as well as CD8+ T-cells showed a distinct increase in the percentage of naïve cells after 5-Aza treatment (Figure 5(a), the gating strategy is depicted in Supplementary Figure S2). Within the CD8+ T-cell compartment, memory effector cells decreased in number, while terminally differentiated effector cells showed a slight increase, suggesting that various subsets react differently to 5-Aza-mediated inhibition.

Intact T-helper cell memory function is required for effective reactivation and stimulation of cytotoxic CD8+ T-cells. We observed a reduction especially in the central memory CD4+ T-cell compartment (Figure 5(a)), confirming that 5-Aza impairs memory function in both major T-cell subpopulations.

**Figure 6:**
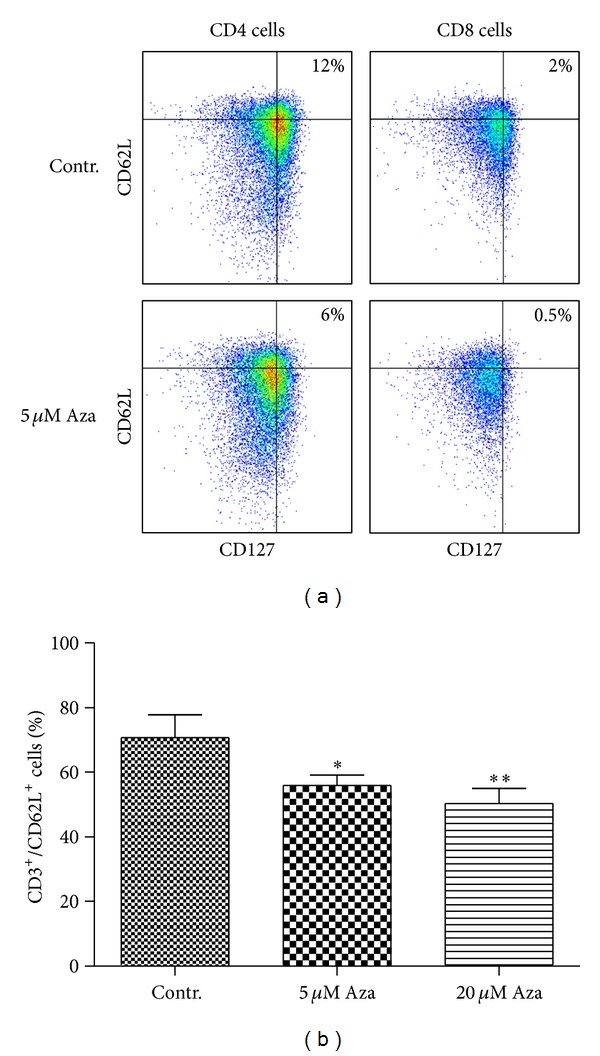
Treatment with 5-Azacytidine reduces long-term memory cell phenotype. (a) CD3+ were treated for 48 h with 5 *μ*M 5-Aza or untreated as control. Thereafter, T-cells were first assessed for their CD4 and CD8 expression. CD4+ T-cells as well as CD8+ T-cells were further analyzed for the expression of CD62L and CD127. High expression (>10^4^ compared to isotype control) of both antigens was taken as a surrogate marker for long-term memory cells. A representative example for three independent experiments is shown. (b) CD3+ T-cells were analyzed for the expression of CD62L by flow cytometry after treatment with 5-Aza. Data were shown as mean value of 3 independent experiments with standard deviation. (*P < 0.05; **P < 0.01).

Memory cells express a range of homing molecules facilitating their migration into lymphoid tissues, such as CD62L [25]. Cells expressing CD62L together with IL7-R*α* have been suggested to represent a long-term memory population [26]. We were able to detect this population in the CD4+ and CD8+ T-cell subset of untreated cells. Investigating the influence of 5-Aza on this population by flow cytometry, we observed a reduction of CD127hiCD62Lhi cells in both compartments (Figure 6(a)). Reduction of CD62L was significant in the overall CD3+ T-cell compartment (*P* < 0.05 and *P* < 0.01; Figure 6(b)).

Taken together, these findings support our hypothesis that 5-Aza interferes with the memory phenotype of T-cells and stabilizes the naïve phenotype.

### 3.6. *In Vivo* Effects of 5-Azacytidine in the Treatment after alloSCT

To evaluate the effects of 5-Aza* in vivo*, we monitored 3 patients who were treated with 5-Aza because of relapse or minimal residual disease after alloSCT. Patient's characteristics were shown in Supplementary Table 1. None of the patients developed a graft versus host disease. As the* in vitro* data suggest that 5-Aza modulates T-cells towards a regulatory phenotype and inhibits proinflammatory T-cells, we monitored our patients for the aforementioned T-cell subpopulations (e.g., CD4+ T-cells, CD8+ T-cells, CD3+/HLA-DR+, Treg, naïve/memory CD4+, and naïve/memory CD8+ T-cells).

**Figure 7:**
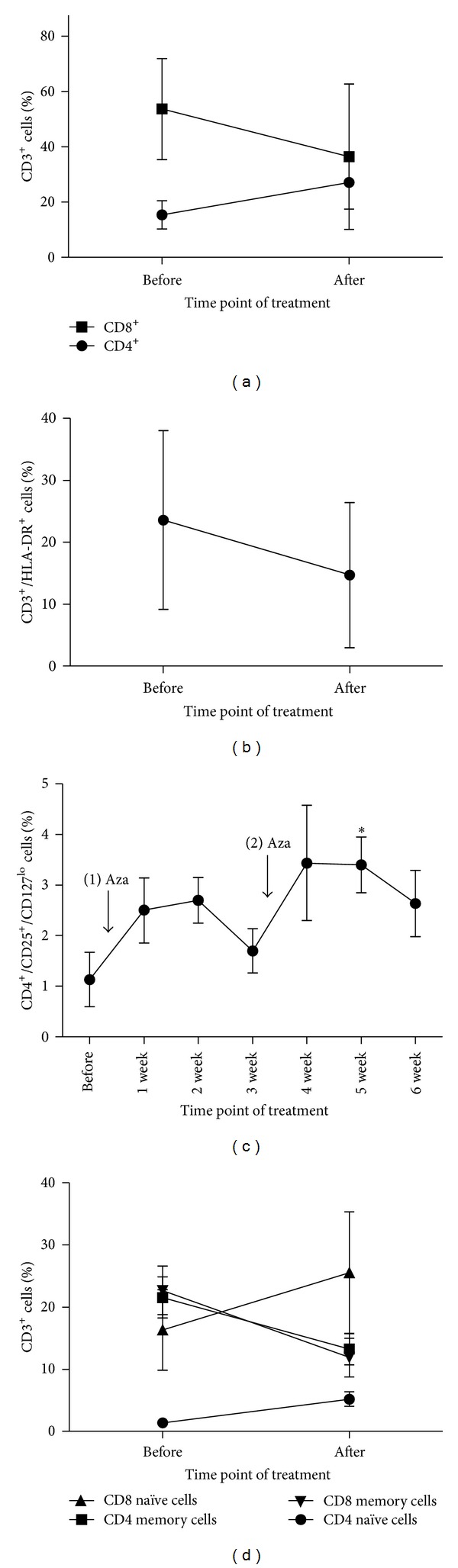
*In vivo* effects of 5-Azacytidine in the treatment after alloSCT. (a) CD4+ and CD8+ T-cells were measured in the peripheral blood of three different patients during their treatment with 5-Aza. Data is shown as mean with SD and depicted values are baseline (before the first application of 5-Aza) and at the end of the 2 cycles (after treatment). (b) CD3+ T-cells were analyzed for the expression of HLA-DR by flow cytometry as long-term activation marker during treatment with 5-Aza. Time points are identical with those in (a). Data were shown as median with SD. (c) Cells expressing CD4+/CD25hi/CD127lo were considered to be Treg cells. Tregs were measured in weekly intervals for both cycles. Data were shown as mean with SD. (↓ shows the time point of 5 days of 5-Aza as treatment). (*P < 0.05). (d) Naïve T-cells were classified by the expression of CD3+/CD4+ or CD8+/CD45RA+. In contrast, cells expressing CD45RO were considered to be memory T-cells. Data is shown as mean with SD and depicted values are baseline (before the first application of 5-Aza) and at the end of the 2 cycles (after treatment).

In accordance with the* in vitro* data of Figure 1(c), we observed an increase of CD4+ T-cells (Figure 7(a) before treatment 15.4% versus 27.1% after 2 cycles of 5-Aza) while in the same time CD8+ T-cells were reduced from 53.3% to 36.3%. The CD8+ T-cell number reduction can be attributed to the upregulation of cell cycle inhibitor* p15*.

Furthermore, one of the main observations of the* in vitro* T-cell cultures was the immune modulating shift of activated proinflammatory T-cells towards a regulatory phenotype (Figures 2, 3, and 4(a)). To investigate the* in vivo* effects of 5-Aza on Tregs and activated T-cells we monitored both populations in our group of patients. While activated T-cells, marked by HLA-DR expression, decreased from 23.5% to 14.5% after 2 cycles of 5-Aza (Figure 7(b)), Tregs showed a more dynamic development (Figure 7(c)). As expected from the* in vitro* data Treg showed an increase after the 5 days of 5-Aza application (1.3% before to 2.5% after the 1 week of treatment). In week 2 of the first cycle of treatment, Treg levels increased slightly to 2.7%, but in week 3 after 5-Aza treatment, Treg-cells started to decrease to 1.7%. After this week, patients received a second application of 5-Aza (100 mg/m^2^ on 5 following days). In the following week, Tregs expanded again up to 3.4% (*P* = 0.58). Treg numbers increased significantly after 5 weeks of treatment (Figure 7(c), 1.3% before versus 3.5% after 5 weeks, *P* < 0.05).

Moreover, our* in vitro* findings suggested that naïve T-cells were less sensitive to 5-Aza-mediated growth inhibition than their memory counterparts. We analyzed the blood of our patients for the development of naïve and memory T-cells during the treatment period. In accordance with our* in vitro* data, we confirmed that naïve T-cells were not inhibited by 5-Aza treatment* in vivo* (Figure 7(d), for CD4 naïve cells 1.0% before to 5.2% after treatment, *P* = 0.05, for naïve CD8 T-cells from 16.3% to 25.5% after treatment). In contrast to naïve cells, memory T-cells were reduced by the treatment of 5-Aza in both major subsets (for memory CD4+ T-cells 21.5% before to 13.2% after treatment, for memory CD8+ T-cells from 22.6% to 11.9% after treatment).

## 4. Discussion 

The demethylating agent 5-Azacytidine is broadly used for the treatment of MDS and some forms of AML and first reports showed that 5-Aza is a safe treatment option after allogeneic stem cell transplantation [9, 10, 27]. In this study we investigated whether treatment with 5-Aza is likely to contribute or detract from the predominantly T-cell mediated antitumor immune response.

One mechanism of action of 5-Aza is to suppress proliferation by upregulation of cell cycle inhibitory genes [5]. We show that* in vitro* treatment of human CD4+ and more extensively CD8+ T-cells results in reduced culture growth, by expression of* p15* located in the INK4 locus, which is predominantly controlled by the DNA methylation of CpG islands [28, 29]. Furthermore, we can confirm this* in vitro* effect* in vivo*, as CD4+ T-cells were increased during 5-Aza treatment of patients, while CD8+ T-cell numbers decreased (Figure 7(a)). We also observed upregulation of* p15* particularly in CD8+ T-cells. Induction of* p15*, a key regulator of G1 phase progression [30], may play a central role in the 5-Aza-mediated growth arrest of T-lymphocytes following 5-Aza treatment. However Goodyear et al. reported that that lower dosages of 5-Aza did not affect the number of CD3+, CD4+, and CD8+ cells* in vivo* [31] which may be explained by the need of a 5 *μ*M dosages to significantly affect the expression of* p15* in CD8+ T-cells* in vitro* (Figure 1(c)).

FOXP3 is the hallmark transcription factor of Tregs and has been shown to be tightly controlled by epigenetic modifications [15, 16]. Strikingly, we found a twofold increase of FOXP3+ Treg cells, following 5-Aza treatment. We showed that regulatory T-cells induced by 5-Aza produce characteristic inhibitory cytokines, like IL-10 and TGF-*β* [32, 33]. However, as expression of both cytokines is also controlled by DNA methylation, an increase may theoretically be the result of either lineage-specific and/or 5-Aza-mediated demethylation [34]. Additionally, we were able to observe an increase of Treg in our group of patients (Figure 7(c)) after the first week of 5-Aza treatment (*P* = 0.1), while after the second week of 5-Aza treatment, Treg numbers increased more (week 4: 3.4% *P* = 0.58 and week 5: 3.4% *P* < 0.05). However, 5-Aza induced Tregs decrease at week 3 and week 6 of treatment, arguing that 5-Aza leads to induction of Treg. Those induced Treg cells are reported to lose their inhibitory function [35] and therefore this inhibitory effect of 5-Aza might be temporal and treatment dependent.

Apart from Tregs, additional populations with regulatory potential coexist with effector populations, in the T cell compartment [36, 37]. Despite its role as the hallmark transcription factor of conventional regulatory T-cells,* FOXP3* expression has also been described in a CD8+ T-cell subset referred to as T-suppressor cells [37]. As anticipated, we observed a strong dose-dependent upregulation of* FOXP3* mRNA in CD8+ T-cells and a shift towards T-suppressor cells, following 5-Aza treatment. These cells share some of the phenotypic characteristics of conventional CD4+ regulatory T-cells [37] but show less IFN-*γ* production following stimulation, even though they have recently been described by minor inhibitory potential [38]. However, we found that treatment with 5-Aza strongly impaired tumor-specific killing by a mixed CD3+ T-lymphocyte population as well as purified cytotoxic CD8+ T-cells. We propose that CD8− restricted and possibly even CD4− T-cell mediated inhibitory signals played only a minor role in our setting and that cell cycle inhibition, preventing expansion of tumor-specific clones and limited IFN-*γ* production, are likely to constitute an explanation of our observations.

Uncompromised lineage-specific differentiation of T-helper subsets is important for the ability of the immune system to develop a relevant antitumor response and may be affected by undirected DNA hypomethylation. The central effector molecule of the proinflammatory Th1 population, IFN-*γ*, is known to be controlled by promoter methylation [39]. However, we did not observe induction, but rather a decrease of IFN-*γ*+ cells as well as a downregulation of Th1 master regulator* TBET1*. Suboptimal 5-Aza dosage might have influenced this result, although our observation of an actual loss of IFN-*γ*+ Th1 cells would argue against this hypothesis and for a targeted shift in T-helper differentiation.* In vivo* experiments in mice and first reports in humans further support the suppressive properties of 5-Aza on CD4+ T-cells, in which 5-Aza treatment was sufficient for engraftment and prevented GvHD occurrence [13, 31]. In addition, while Th2 cells appeared to remain stable under 5-Aza treatment, we did notice a slight increase in IL-17 producing proinflammatory Th17 cells, theoretically arguing against a generally immunosuppressive role of 5-Aza as described up to this point. Interestingly, the increase of Th17 cells, despite the threefold exceeding therapeutic dose of 5-Aza, was comparably minor and may have only limited functional consequences. Moreover, considering the global mode of action of 5-Aza and strong modulation of subset composition the possibility of the coexpression of several cytokines, as recently described in multicytokine producing cells [40], may need further investigation. Despite the possible proinflammatory effects that may occur in small T-subcell groups we did not observe a case of GvHD in our small group of patients, which is in accordance with published data, reporting induction of GvHD as a rare event during 5-Aza treatment, even in combination with donor lymphocytes infusions (DLI) [9]. This observation argues against a dominant proinflammatory nature of 5-Aza on T-cells and favours the, here reported, more regulatory character of the drug as suggested by a murine* in vivo* model [13]. Further, most of the effects on cytokine upregulation and induction of regulatory T-cell subsets were observed with 5 *μ*M 5-Aza, a dosage which is close to that which is reached with 75 mg/m^2^ in the clinical setting [41].

In a more resent publication by Costantini et al. the authors also reported that Treg cells were upregulated and Th1 cells were decreased. However, FOXP3+ T-cells were reported to lack their suppressive and proliferation capacity [42]. One difference is that Costantini and Coworkers used 5-Aza much more frequently (e.g., every 24 h) in comparison to our experimental setup (every 48 h). Further we stimulated our cells with IL2, a cytokine known to be important for Treg survival while Costantini and colleagues only used CD3/CD28 stimulation. This experimental setup may explain some of the difference in the proliferation of Treg cells* in vitro*. Of note, Costantit also reported that the Th17-cells were rather unaffected by 5-Aza treatment [42].

Due to our findings indicating a differentiation capacity of 5-Aza especially in Treg and Th1 cells, we further analyzed its impact on the naïve and memory T-populations required for an effective antitumor immunity [43]. Our data indicate that the increase of naïve cells, in both major T-cell compartments, is caused by a higher resistance of naïve T-cells against 5-Aza-mediated cell cycle inhibition. This* in vitro *effect could be confirmed by our* in vivo* data, as indicated in the observed increase of both naïve cell subsets in all patients (Figure 7(d)). Memory T-cells have been reported to exhibit higher responsiveness to pro-proliferative signalling, suggesting a more dynamic control of cell cycle checkpoints. 5-Aza might be more likely to interfere with cell cycle progression in cells susceptible to respond to proliferative stimuli, explaining the relative reduction of memory T-cells under 5-Aza treatment* in vivo*. Long-term memory cells, as recently characterized by CD62LhiCD127hi immunophenotyping [26], were significantly reduced after 5-Aza treatment. However, this population's classification and tumor-specific lytic capacity requires further investigation* in vivo*.

Taken together, we have shown that 5-Aza treatment variably alters the profile of adaptive T-cell immunity. The shift from cytotoxic to regulatory T-cells with a functional phenotype and the major reduction of proinflammatory Th1 cells, as well as effector memory T-cells, all indicate a strong inhibition of T-cell function by 5-Aza. This is further supported by CD8+ T-cells adopting a growth-delayed FOXP3+ phenotype, consistent with reduced cytotoxic activity including impaired IFN-*γ* production. Overall, our data indicate that 5-Aza treatment, while theoretically enhancing antitumor immunity by inducing the expression of tumor antigens, can significantly affect the adaptive cellular response, by promoting an inhibitory T-cell phenotype.

## Supplementary Material

Flow cytometric analysis of intracellular granzyme and analysis of naïve cells T-cells were purified and kept as described. T-cells were treated with the mentioned concentrations of 5-Aza.
For the analysis of intracellular proteins T- cells were stimulated with phorbol myristate acetate (PMA), Ionomycin for 1 hour and Brefeldin A for 4.5 hours. All chemicals were obtained from Sigma-Aldrich (Munich, Germany). Cells were harvested and prepared for analysis using the Cytofix/Cytoperm kit (BD Bioscience, Heidelberg, Germany). For intracellular cell staining the following antibody was used: anti-Granzyme-PE. Prior to intracellular staining cells were marked with anti-CD4-FITC, and anti-CD8-APC (all antibodies obtained from BD Bioscience, Heidelberg, Germany).
For analysis of naïve T-cells, cells were stained after Aza treatment with the following antibodies: anti-CD4- PerCP, anti-CD8-APC, anti-CCR7-PE, and anti- CD45RA-FITC (all antibodies obtained from BD Bioscience, Heidelberg, Germany).
All analysis were performed using a Canto II (BD Bioscience, Heidelberg, Germany) and data were further analysed using the FlowJo Software (TreeStar Inc, Ashland, USA).
