# Supplementary material for: 5-Azacytidine Promotes an Inhibitory T-Cell Phenotype and Impairs Immune Mediated Antileukemic Activity
Source: Mediators Inflamm. 2014 Mar 13;2014:418292. doi: 10.1155/2014/418292 (PMC3976863; doi:10.1155/2014/418292)
Supplement: Supplementary file 1 — Flow cytometric analysis of intracellular granzyme and analysis of naïve cells T-cells were purified and kept as described. T-cells were treated with the mentioned concentrations of 5-Aza. For the analysis of intracellular proteins T- cells were stimulated with phorbol myristate acetate (PMA), Ionomycin for 1 hour and Brefeldin A for 4.5 hours. All chemicals were obtained from Sigma-Aldrich (Munich, Germany). Cells were harvested and prepared for analysis using the Cytofix/Cytoperm kit (BD Bioscience, Heidelberg, Germany). For intracellular cell staining the following antibody was used: anti-Granzyme-PE. Prior to intracellular staining cells were marked with anti-CD4-FITC, and anti-CD8-APC (all antibodies obtained from BD Bioscience, Heidelberg, Germany). For analysis of naïve T-cells, cells were stained after Aza treatment with the following antibodies: anti-CD4- PerCP, anti-CD8-APC, anti-CCR7-PE, and anti- CD45RA-FITC (all antibodies obtained from BD Bioscience, Heidelberg, Germany). All analysis were performed using a Canto II (BD Bioscience, Heidelberg, Germany) and data were further analysed using the FlowJo Software (TreeStar Inc, Ashland, USA). [file 418292.f1.pdf]

Supplementary Figure 1

Figure Legend

Figure S1. CD3+ T- cells were treated with 5µM, 20µM or without 5-Aza for 48h. Then cells were stimulated with PMA and ionomycin in the presence of brevedinA for 4.5h. Thereafter T- cells were analysed for the expression of CD4 and CD8. The CD8+ T-cells were further assessed for the production of granzyme. A representative FACS pot of three independent experiments is shown.

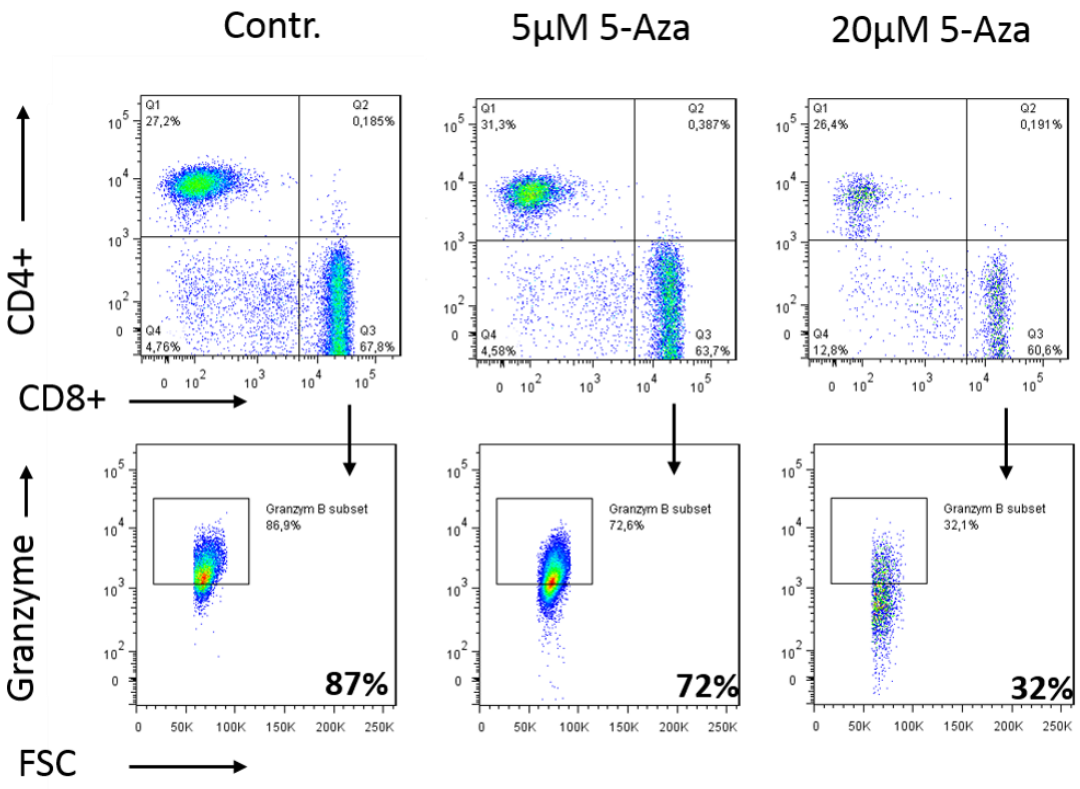

Supplementary Figure 2.

Figure Legend

Figure S2. Gating strategy for identification of naïve T- cells. T- cells were cultured for 48h with the indicated dosages of 5-Aza. Untreated T- cells were used as control. Cells were analysed for the expression of CD4 to identify CD4+ T- cells which were further sub gated for the expression of CD45RA and CCR7.Cells expressing both (CD45RA+/CCR7+) were considered to be naïve T- cells. The same strategy was used to identify naïve CD8+ T- cells.

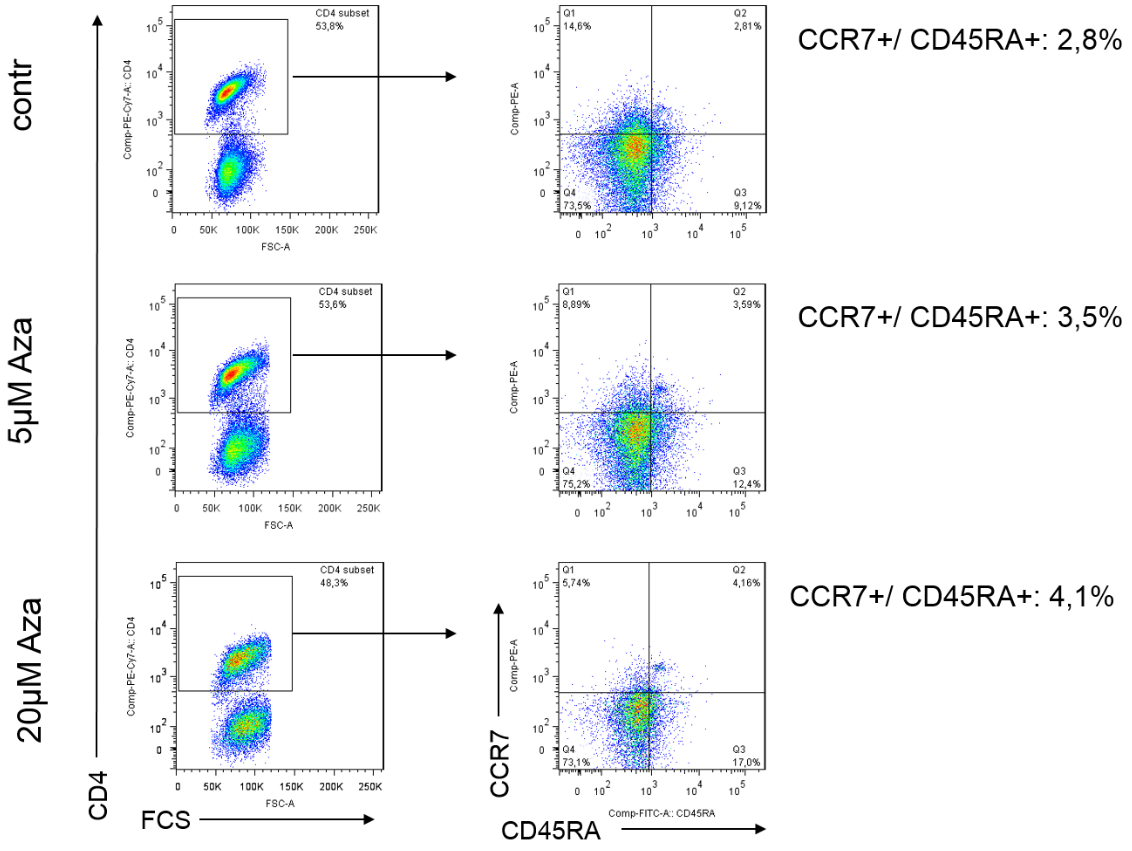

Supplementary Table 1

Patient	Age	Sex	Disease	Cytogenetic molecular genetics	Conditioning	Stem cell source	Outcome	5-Aza start
1	63y	M	sAML	Tri8, CBL mut	TBI / Flu	PBSC	death	+ 66 d
2	57y	M	CMML	nk, TET2 mut, CBL mut	FLAMSA/ Bu Cy	PBSC	alive	+ 96 d
3	61y	M	MDS/MPN	nk NRAS mut	Bu / Flu	PBSC	alive	+ 127 d

Abbreviations: (M) male, (sAML) secundary actue myeloblastic leukemia, (CMML) chronic myelomonocytic leukemia, (MDS/MPN) myelodysplastic syndrome, myeloproliferative neoplasme. (Tri8) trisomie of chromosome 8, (nk) normal caryotype, (NRAS mut) NRAS mutation codon 61, (CBL mut) CAS-BR-M murine ecotropic retroviral transforming sequence homolog mutation, (TET2 mut) TET oncogene family member 2 mutation, (FLAMSA) Fludarabine, mAmsacrine, Cytarbine, (Bu) Busulphan, (Cy) Cyclophosphamide, (TBI) total body irradiation, (Flu) Fludarabine , (PBSC) peripheral blood stem cells.
